# Increased diet-induced fatty streak formation in female mice with deficiency of liver-derived insulin-like growth factor-I

**DOI:** 10.1007/s12020-015-0809-1

**Published:** 2015-12-01

**Authors:** Johan Svensson, Klara Sjögren, Malin Levin, Jan Borén, Åsa Tivesten, Claes Ohlsson

**Affiliations:** Centre for Bone and Arthritis Research, Institute of Medicine, Sahlgrenska Academy, University of Gothenburg, 413 45 Göteborg, Sweden; Department of Molecular and Clinical Medicine/Wallenberg Laboratory, Institute of Medicine, Sahlgrenska Academy, University of Gothenburg, 413 45 Göteborg, Sweden; Department of Internal Medicine, Sahlgrenska University Hospital, Gröna Stråket 8, 413 45 Göteborg, Sweden

**Keywords:** IGF-I, Liver derived, Fatty streak, Paigen diet, Inflammation, Endothelial dysfunction

## Abstract

The role of endocrine IGF-I for atherosclerosis is unclear. We determined the importance of circulating, liver-derived IGF-I for fatty streak formation in mice. Mice with adult, liver-specific IGF-I inactivation (LI-IGF-I^−/−^ mice, serum IGF-I reduced by approximately 80 %) and control mice received an atherogenic (modified Paigen) diet between 6 and 12 months of age. At study end, Oil Red O staining of aortic root cryosections showed increased fatty streak area and lipid deposition in female but not in male LI-IGF-I^−/−^ mice compared to controls. Mac-2 staining of aortic root and measurements of CD68 mRNA level in femoral artery revealed increased macrophage accumulation in proportion to the increased fatty streak area in female LI-IGF-I^−/−^ mice. Moreover, female LI-IGF-I^−/−^ mice displayed increased serum cholesterol and interleukin-6 as well as increased vascular cell-adhesion molecule 1 (VCAM1) mRNA levels in the femoral artery and elevated VCAM1 protein expression in the aortic root. Thus, increased diet-induced fatty streak formation in female LI-IGF-I^−/−^ mice was associated with increased serum cholesterol and signs of systemic inflammation, endothelial activation, lipid deposition, and macrophage infiltration in the vascular wall.

## Introduction

Insulin-like growth factor-I (IGF-I) is important for cell growth and proliferation [1]. Circulating IGF-I, which is mainly liver-derived [1–3], gradually declines with increasing age and is dependent on growth hormone (GH) secretion, food intake, and exercise [1]. Experimental studies suggest that in addition to regulating postnatal longitudinal growth, IGF-I maintains brain function, cortical bone mass, cardiovascular performance, and metabolic indices during adulthood [1].

There is increasing clinical evidence that low IGF-I is a risk factor for cardiovascular disease (CVD) morbidity. Studies of polymorphisms in the IGF-I gene demonstrate a link between low serum IGF-I levels and impaired measures of early atherosclerosis such as increased carotid intima-media thickness [4, 5]. Hypopituitary patients with GH deficiency and secondary low circulating IGF-I have increased CVD morbidity and mortality [6–8]. Epidemiological studies have shown an association between low serum IGF-I concentrations and increased risk of ischemic heart disease [9], congestive heart failure [10], and CVD mortality [11, 12]. A study of elderly Swedish men demonstrated that not only low but also high serum IGF-I associates with increased risk of CVD, suggesting a non-linear U-shaped relation between serum IGF-I and CVD risk [13]. In chronic heart failure, patients with low serum IGF-I had increased all-cause mortality compared to those with higher levels [14]. Notably, GH may exert direct effects on vascular cells [1, 15], and the importance of possible alterations in GH secretion has not been evaluated in previous studies.

In experimental models, the role of IGF-I in the development of atherosclerosis has been controversial. Several studies have indicated a permissive role of IGF-I in atherosclerosis [16, 17], particularly by inducing vascular smooth muscle cell (VSMC) migration and proliferation [18, 19]. However, the stimulatory effects of IGF-I on VSMCs might result in stabilization of plaques as supported by experiments performed both in vitro and in vivo [20]. Furthermore, local smooth muscle-specific IGF-1 overexpression did not alter total atherosclerotic plaque burden but resulted in a more stable atherosclerotic lesion phenotype [21]. Pharmacological administration of IGF-I by systemic infusions reduced atherosclerosis progression [22, 23], and genetically modified mice with a 20 % reduction of circulating IGF-I displayed increased atherosclerosis [24]. Finally, pharmacological IGF-I administration may reduce vascular inflammation [22] by improving nitric oxide (NO) availability and reducing oxidative stress [22, 25]. In summary, the role of IGF-I for atherosclerosis development is still rather unclear and the specific role of liver-derived circulating IGF-I has not yet been evaluated.

In the present study, we determined the role of liver-derived circulating IGF-I for diet-induced fatty streak formation in adult mice using an inducible mouse model with selective inactivation of the IGF-I gene in the liver (LI-IGF-I^−/−^ mice). The LI-IGF-I^−/−^ mice display an approximately 80 % reduction of serum IGF-I concentrations, whereas the expression of IGF-I is not reduced in other tissues [1, 2].

## Materials and methods

### Animals and serum IGF-I

The LI-IGF-I^−/−^ mice on a C57BL/6 background were generated as described previously [1, 2]. Mice homozygous for LoxP [26] and hemizygous for Mx-Cre [27] received three ip injections of polyinosinic-polycytidylic acid (PiPc; 6.25 µg/g body weight; Sigma-Aldrich Corp., Stockholm, Sweden) at 3 months of age to induce expression of the Cre protein, thereby inactivating the IGF-I gene in the liver [27]. PiPc-treated female and male littermates, homozygous for LoxP but lacking Mx-Cre, were used as controls as previously described [2, 3, 26]. Seven days after the PiPc injections as well as at the end of the study, serum was obtained and assayed for IGF-I by a double-antibody IGF-binding protein-blocked RIA (Mediagnost, Tübingen, Germany). The mice were housed in a standard animal facility under controlled temperature (22 °C) and photoperiod (12 h of light, 12 h of dark) with free access to water and food pellets (B&K Universal AB, Sollentuna, Sweden). Animal care was in accordance with institutional guidelines. The ethical committee at the University of Gothenburg approved this study.

### Study protocol

All mice (female mice: control, *n* = 13; LI-IGF-I^−/−^, *n* = 9; male mice: control, *n* = 16; LI-IGF-I^−/−^, *n* = 15) received an atherogenic diet (modified Paigen) diet between 6 and 12 months of age. The modified Paigen diet (diet 12336, Research Diets, Inc., New Brunswick, NJ, USA) was made of purified components designed to match the original Paigen diet [28]. The caloric composition was 20 % protein, 35 % fat, and 45 % carbohydrate, and the diet contained 1.25 % cholesterol and 0.5 % cholate.

Blood was drawn from vena saphena magna before and 2 weeks after the start of the atherogenic diet. After 6 months of the modified Paigen diet (at 12 months of age), dual-energy X-ray absorptiometry (DEXA) was performed under anesthesia with fentanyl and fluanisone (0.55 and 17.5 mg/kg; Hypnorm, Janssen Pharmaceuticals, Beerse, Belgium) and midazolam (8.75 mg/kg; Dormicum, Hoffman-La-Roche Inc., Basel, Switzerland). The circulatory system was then perfused with 0.9 % saline (pH 7.4) under physiological pressure. The aortic root was slowly frozen in optimum cutting temperature embedding medium (Sakura Tissue-Tek, Tokyo, Japan). Tissue from the femoral artery was snap frozen in liquid nitrogen for later analysis using RT-PCR.

### Analysis of serum lipids and cytokines

We used infinity reagents (cholesterol no. TR13421 and triglycerides no. TR22421; Thermo Fisher Scientific, Pittsburgh, PA, USA) to analyze total cholesterol and triglycerides in individual serum samples. Cytokine levels were measured using a cytokine multiplex panel (mouse pro inflammatory Vplex from Meso Scale Discovery, Gaithersburg, MD, USA), including interferon (IFN)-γ, interleukin (IL)-1β, IL-2, IL-5, IL-6, keratinocyte-derived chemokine (KC/GRO), and tumor necrosis factor (TNF)-α, in individual serum samples using the electro-chemiluminescence multiplex system Sector 2400 imager (Meso Scale Discovery).

### DEXA

Total body fat and total body lean mass were measured in vivo at the end of the 6-month modified Paigen diet using dual-energy X-ray absorptiometry (DEXA; PIXImus, Lunar Corporation, Madison, MI, USA). The DEXA scans were from the lower level of the head to the inferior level of the first tail vertebra (the maximal scan area of the DEXA).

### Lesion analyses in the aortic root

Serial 10-μm cryosections were cut distally from the aortic root. The sections immediately after the appearance of the aortic cusps (i.e., at 0 μm) were stained with Oil Red O (Sigma-Aldrich, St. Louis, MO, USA). For immunohistochemical staining of macrophages, aortic root cryosections (40 μm from the aortic cusps) were incubated with rat anti-mouse Mac-2 antibody (1:1000; Cedarlane, Hornby, Canada) or isotype control antibody (1:1000; Biolegend, San Diego, CA, USA) followed by horseradish peroxidase (HRP)-conjugated goat anti-rat IgG secondary antibody (1:1000; GE Healthcare, London, UK) and visualized with DAB substrate kit (Dako, Glostrup, Denmark).

All evaluations of aortic root sections were performed by a blinded observer, using morphometric analysis (BioPix Software, Gothenburg, Sweden). Fatty streak area was defined manually and normalized to the area of the vessel wall. The areas of Oil Red O staining and Mac-2 staining were quantified using BioPix Software and normalized to the vessel wall area or the size of the lesion.

For immunohistochemical staining of vascular cell-adhesion molecule 1 (VCAM1), aortic root cryosections (80 μm from the aortic cusps) were incubated with anti VCAM1 antibody (1:50; BD Pharmingen, San Diego, CA, USA) or isotype control antibody (1:50; BD Bioscience, Franklin Lakes, NJ, USA) and quantified using software from Visiopharm (Hoersholm, Denmark).

### Triglyceride content in liver

Liver samples were homogenized with isopropanol using stainless steel beads (5 mm; Qiagen, Hilden, Germany) in a Tissue-Lyser II (Qiagen), kept for 1 h at 4 °C, and centrifuged for 5 min (2500 rpm, 4 °C), and the supernatants were collected. The triglyceride levels in the supernatants were analyzed using an Infinity reagent (Triglycerides #TR22421; Thermo Fisher Scientific), and the amount of triglycerides was normalized to liver sample weights.

### Real-time PCR (RT-PCR) analysis

RNA was prepared from the femoral artery using the RNeasy kit (#74106, Qiagen) according to the manufacturer’s instructions. The RNA was reverse transcribed into cDNA using High-capacity cDNA Reverse transcription kit (#4368814, Applied Biosystems, Stockholm, Sweden). RT-PCR analyses were performed using the ABI Prism 7000 Sequence Detection System (PE Applied Biosystems, Carlsbad, CA, USA). We used pre-designed RT-PCR assays from Applied Biosystems for the analysis of CD68 (Mm03047340_ml), vascular cell-adhesion molecule 1 (VCAM1; Mm01320970_ml), endothelial NO-synthase (eNOS; Mm00435217_m1), and IGF-I (Mm00439559_m1) expression levels (Applied Biosystems). The mRNA abundance of each gene was adjusted for the expression of 18S rRNA.

### Statistical analyses

All descriptive statistical results are presented as the mean (SEM). All analyses compared two groups (female LI-IGF-I^−/−^ mice vs. female control mice or male LI-IGF-I^−/−^ mice vs. male control mice) at one timepoint. The between-group differences were calculated using unpaired* t* tests. A two-tailed *p* < 0.05 was considered significant.

## Results

Liver-specific inactivation of the IGF-I gene at 3 months of age reduced serum IGF-I by 81 and 77 % in male and female mice, respectively [male mice: mean (SEM) 202 (6) ng/ml in controls vs. 39 (4) ng/ml in LI-IGF-I^−/−^ mice, *p* < 0.001; female mice: 216 (11) ng/ml in controls vs. 50 (5) ng/ml in LI-IGF-I^−/−^ mice, *p* < 0.001]. At study end, at 12 months of age, serum IGF-I was reduced by 79 and 72 % in male and female mice, respectively [male mice: 238 (14) ng/ml in controls vs. 51 (5) ng/ml in LI-IGF-I^−/−^ mice, *p* < 0.001; female mice: 246 (17) ng/ml in controls vs. 68 (11) ng/ml in LI-IGF-I^−/−^ mice, *p* < 0.001].

### Increased aortic root fatty streak formation in female, but not male, LI-IGF-I^−/−^ mice

**Fig. 1:**
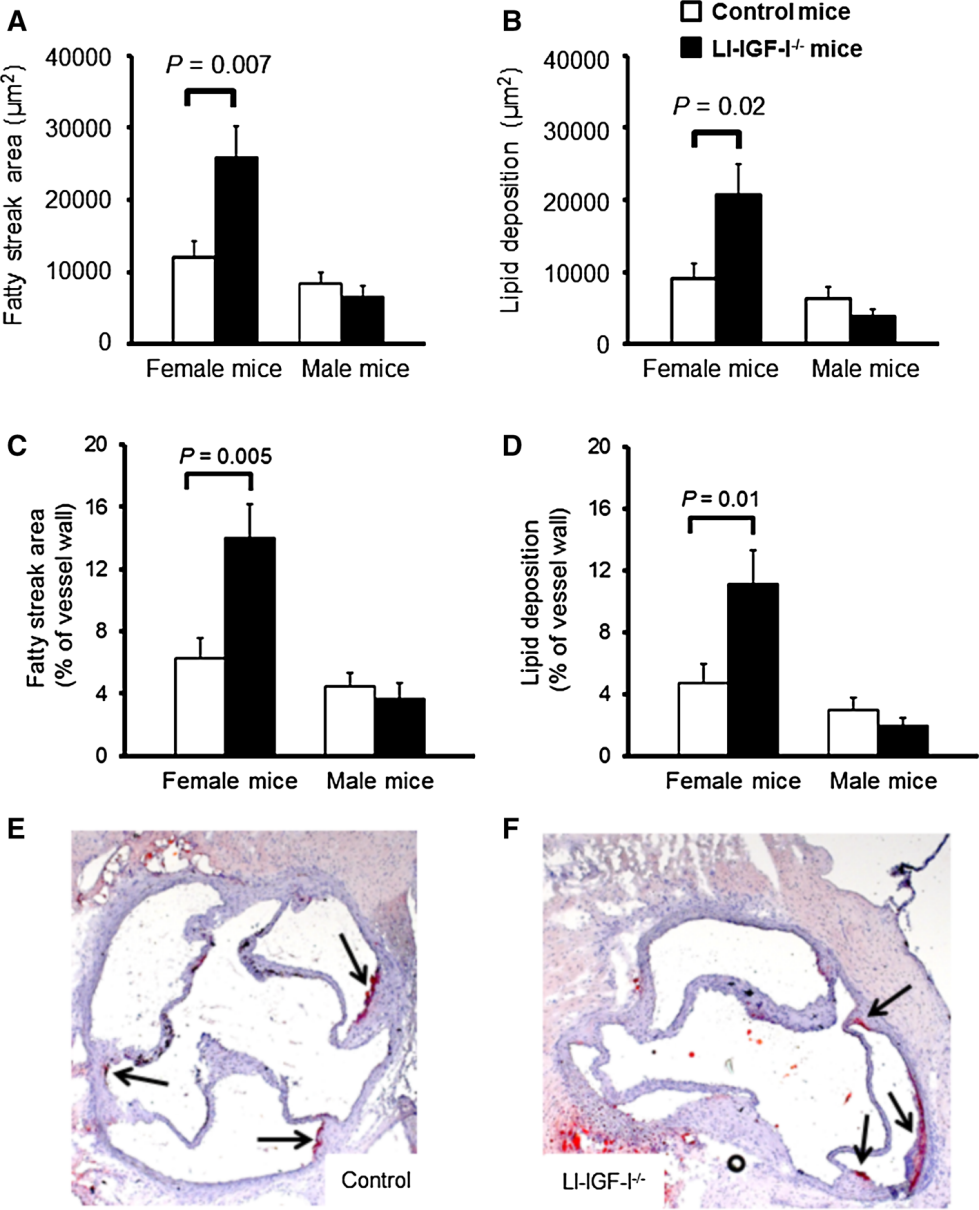
Increased fatty streak formation in aortic wall of female LI-IGF-I^−/−^ mice. Male and female LI-IGF-I^−/−^ and control mice (*n* = 9–16/group) received an atherogenic (modified Paigen) diet from 6 months of age. At the end of the study (12 months of age), **a** fatty streak area and **b** lipid deposition were quantified in Oil Red O-stained aortic root cryosections. **c**, **d** Fatty streak area and lipid deposition are given as % of aortic wall area. One section per mouse was analyzed at the level of the aortic cusps. **e**, **f** Representative photomicrographs from female **e** control and **f** LI-IGF-I^−/−^ mice are shown. Values in **a**–**d** are given as means (SEM). The analyses of fatty streaks and lipid deposition were performed in all mice. *p* values were calculated using unpaired *t* tests. In **e** and **f**, *arrows* point at fatty streaks

Fatty streak formation was evaluated in the aortic root. After 6 months of modified Paigen diet, fatty streak area and lipid deposition (expressed as µm^2^ or % of aortic wall area) were increased in female LI-IGF-I^−/−^ mice compared to those in controls (Fig. 1a–f). In contrast, there were no significant differences in these variables in male mice (Fig. 1a–d). Therefore, since increased fatty streak formation was present only in female LI-IGF-I^−/−^ mice, we performed further characterization of vascular lesions only in female mice.

### Increased aortic root macrophage content in female LI-IGF-I^−/−^ mice

**Fig. 2:**
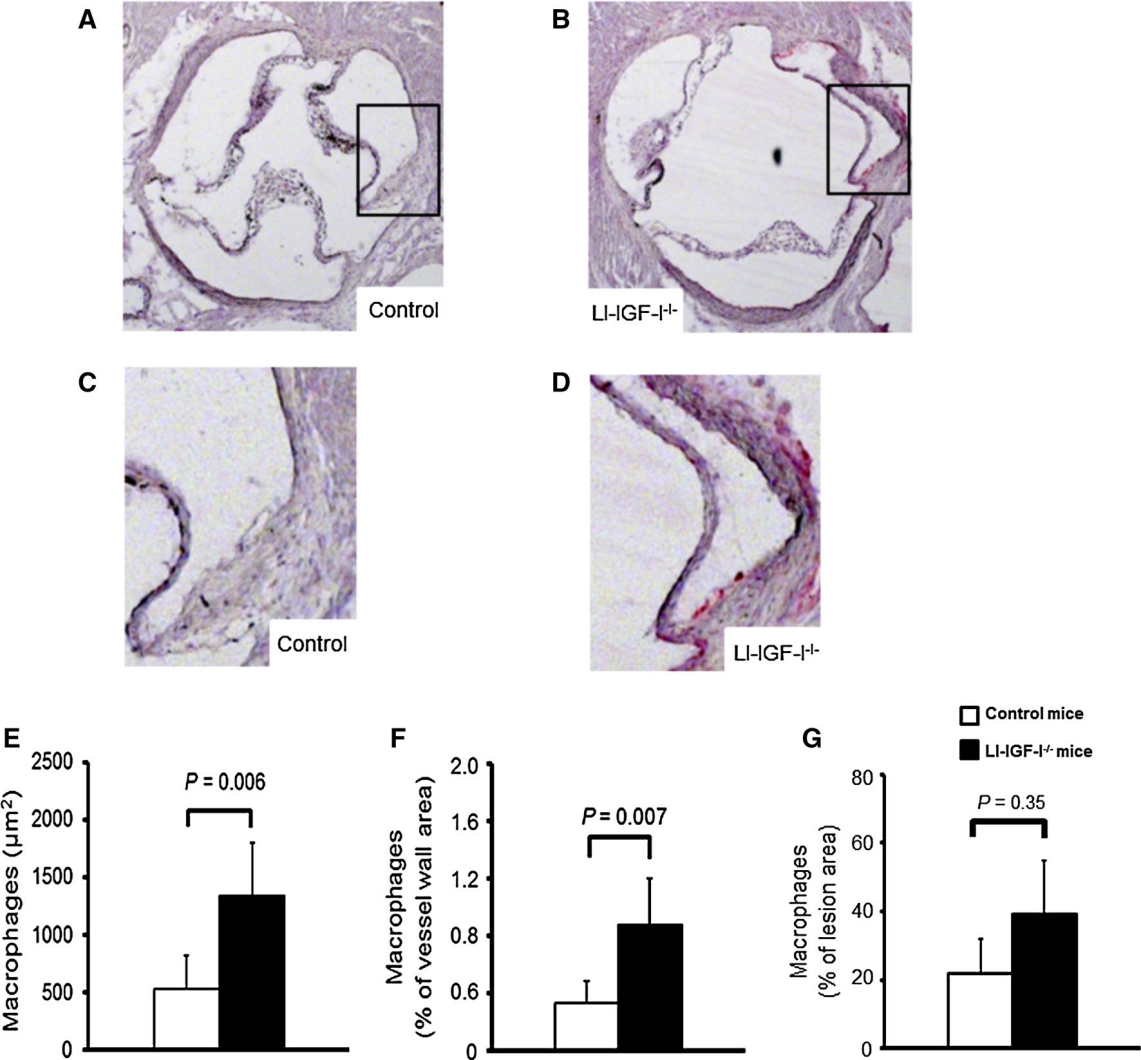
Increased macrophage content in aortic wall of female LI-IGF-I^−/−^ mice. Female control (*n* = 13) and LI-IGF-I^−/−^ (*n* = 9) mice received an atherogenic (modified Paigen) diet from 6 to 12 months of age. Macrophage area was quantified using immunostaining for Mac-2 in aortic root cryosections (40 µm after the aortic cusps). **a**–**d** Representative photomicrographs from female **a**, **c** control and **b**, **d** LI-IGF-I^−/−^ mice are shown. *Areas in the rectangles* of **a** and **b** are enlarged in **c** and **d**, respectively. **e**, **f** Quantification of macrophage area in aortic wall of female mice given as **e** µm^2^ and as **f** % of aortic wall area. **g** Macrophage area was not significantly altered when normalized to the fatty streak area. Values in **e**–**g** are given as means (SEM). *p* values were calculated using unpaired *t* tests

In the female LI-IGF-I^−/−^ mice, vascular macrophage infiltration following the diet period was determined using immunostaining for Mac-2 in the aortic root. The immunostaining for Mac-2 demonstrated that macrophage area as well as macrophage area normalized to the area of the aortic wall was increased (Fig. 2a–f), whereas macrophage area was not significantly altered when normalized to the fatty streak area (Fig. 2g). This suggests mainly unchanged relative macrophage content of fatty streaks in the female LI-IGF-I^−/−^ mice.

### Increased aortic root VCAM1 protein expression in female LI-IGF-I^−/−^ mice

**Fig. 3:**
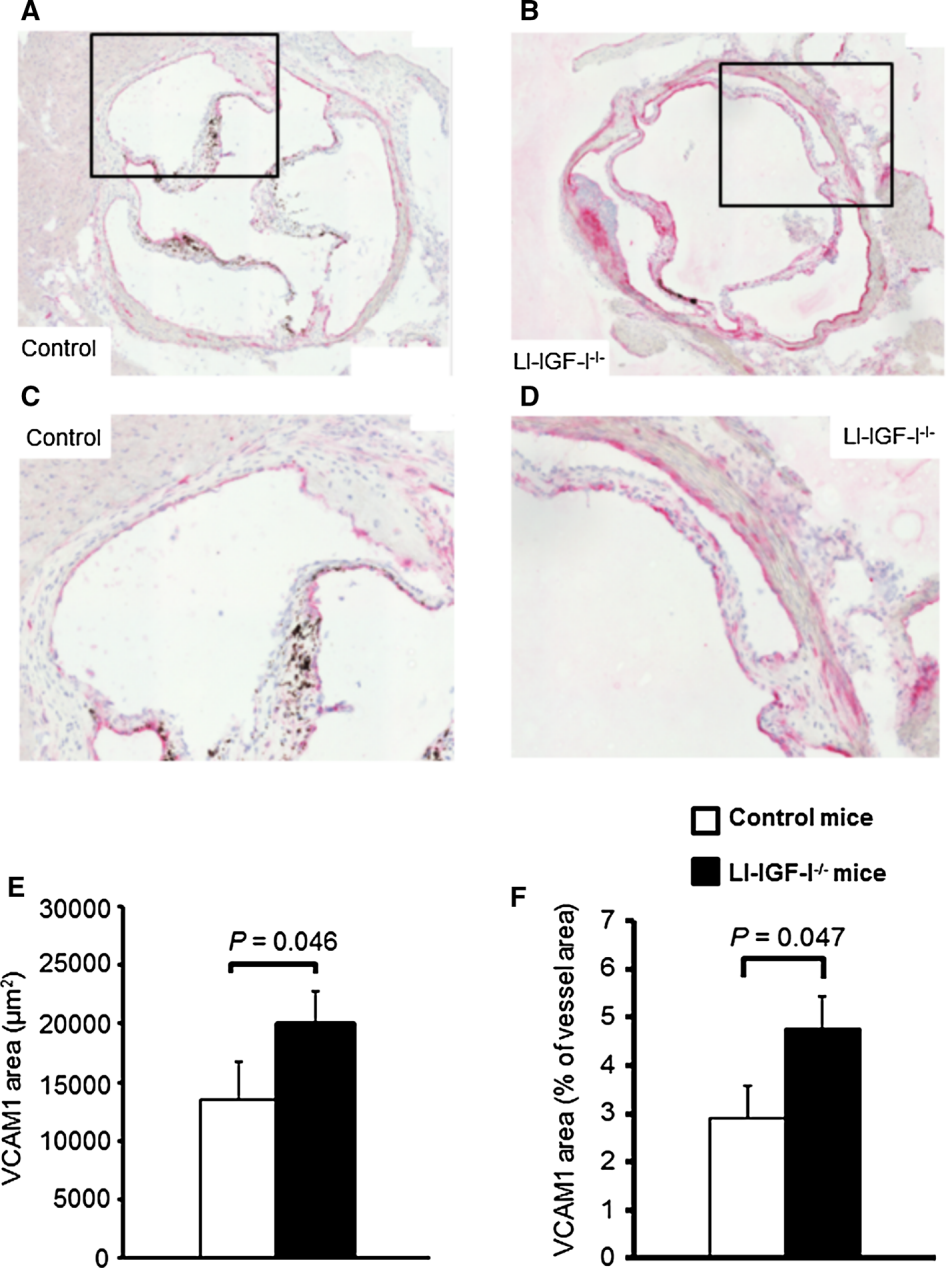
Increased VCAM1 protein expression in aortic root of female LI-IGF-I^−/−^ mice. Female control (*n* = 13) and LI-IGF-I^−/−^ (*n* = 9) mice received an atherogenic (modified Paigen) diet from 6 to 12 months of age. VCAM1 immunostaining was performed in the aortic root (80 µm after the aortic cusps). **a**–**d** Representative photomicrographs from female **a**, **c** control and **b**, **d** LI-IGF-I^−/−^ mice are shown. *Areas in the rectangles* of **a** and **b** are enlarged in **c** and **d**, respectively. **e**, **f** Quantification of VCAM1 immunohistochemistry in aortic wall of female mice given as **e** µm^2^ and as **f** % of aortic wall area. Values in **e**, **f** are given as means (SEM). *p* values were calculated using unpaired *t* tests

As IGF-I affects endothelial function [1], we hypothesized that deficiency of liver-derived IGF-I may result in signs of endothelial dysfunction/activation. Immunohistochemistry analyses revealed increased VCAM1 protein expression in the aortic root of female LI-IGF-I^−/−^ mice (Fig. 3a–f), possibly suggesting endothelial dysfunction/activation in female LI-IGF-I^−/−^ mice.

### Measurements of mRNA levels in femoral artery

**Fig. 4:**
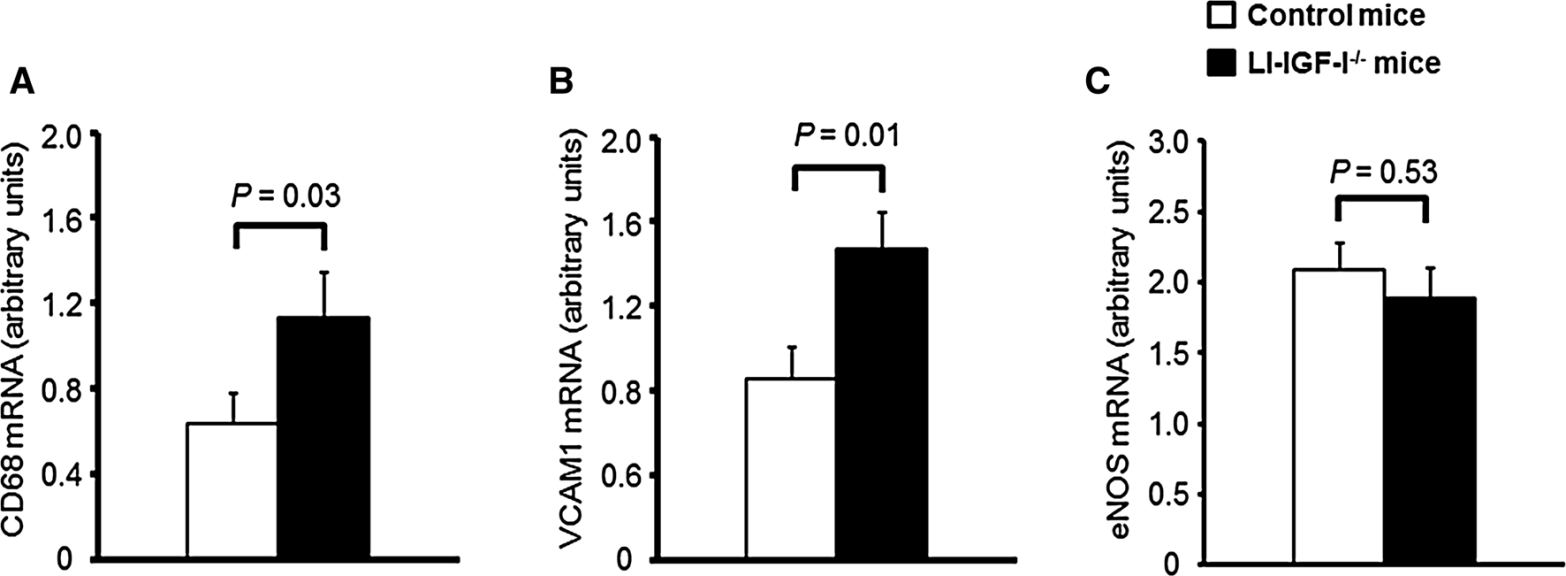
Increased mRNA levels of the macrophage marker CD68 and VCAM1 in femoral artery of female LI-IGF-I^−/−^ mice. Female control (*n* = 13) and LI-IGF-I^−/−^ (*n* = 9) mice received an atherogenic (modified Paigen) diet from 6 to 12 months of age. At study end, mRNA levels of **a** CD68 and **b** VCAM1 were increased in femoral artery of female LI-IGF-I^−/−^ mice. In contrast, **c** eNOS mRNA levels in femoral artery were unchanged in female LI-IGF-I^−/−^ mice compared to control mice. Values are given as means (SEM). *p* values were calculated using unpaired *t* tests

In the femoral artery of female mice, we performed mRNA measurements of the macrophage marker CD68 as well as VCAM1. Furthermore, as IGF-I may improve NO availability [22, 25], we also measured eNOS mRNA levels. The measurements demonstrated elevated mRNA expressions of CD68 and VCAM1 in the femoral artery of female LI-IGF-I^−/−^ mice compared to those of control mice (Fig. 4a, b). However, eNOS mRNA levels in the femoral artery were not significantly altered in female LI-IGF-I^−/−^ mice, to some extent arguing against a NO-mediated mechanism (Fig. 4c).

**Fig. 5:**
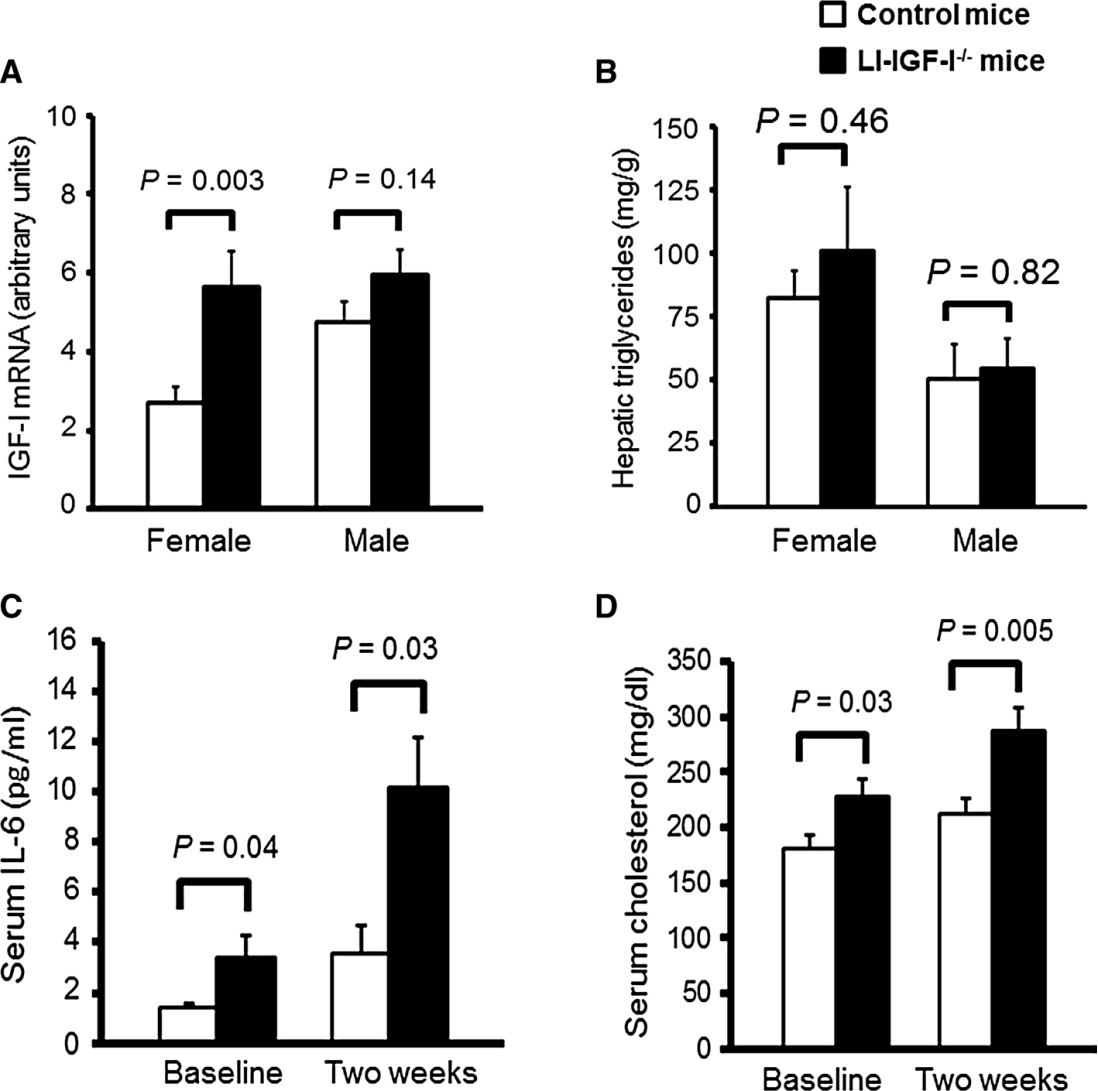
Increased IGF-I mRNA levels in femoral artery and elevated serum IL-6 and cholesterol concentrations in female LI-IGF-I^−/−^ mice. Male and female LI-IGF-I^−/−^ and control mice (*n* = 9–16/group) received an atherogenic (modified Paigen) diet from 6 months of age. **a** IGF-I mRNA levels in femoral artery and **b** hepatic triglyceride content at the end of the study in female and male mice. **c** IL-6, and **d** cholesterol, in serum before the start of the modified Paigen diet (baseline) and after 2 weeks of the diet in female mice. Values are given as means (SEM). *p* values were calculated using unpaired *t* tests

We measured IGF-I mRNA levels in the femoral artery of both female and male LI-IGF-I^−/−^ mice. IGF-I mRNA levels were increased in female LI-IGF-I^−/−^ mice compared to control mice, whereas there was only a non-significant tendency to higher IGF-I mRNA levels in male LI-IGF-I^−/−^ mice (Fig. 5a).

### Hepatic triglyceride content

We measured hepatic triglyceride content as a measure of liver steatosis. However, hepatic triglyceride content was similar in both female and male LI-IGF-I^−/−^ mice compared to that in control mice (Fig. 5b).

### Systemic inflammation in female LI-IGF-I^−/−^ mice

To investigate whether the increased fatty streak area in aortic root of female LI-IGF-I^−/−^ mice was associated with systemic inflammation, serum levels of cytokines were measured. Serum IL-6 concentrations were increased both before and 2 weeks after the start of the atherogenic diet (Fig. 5c) whereas no significant difference was seen for serum levels of IFN-γ, IL-1β, IL-2, IL-5, KC/GRO, and TNF-α.

### Serum lipids and body composition in female and male LI-IGF-I^−/−^ mice

We evaluated serum lipids and body composition in female as well as in male mice. Both before and 2 weeks after the start of the modified Paigen diet, serum cholesterol concentrations (Fig. 5d) but not triglyceride concentrations (+16 and +13 %, both *p* = N.S.; data not shown) were increased in female LI-IGF-I^−/−^ mice compared to those in control mice. In contrast, male LI-IGF-I^−/−^ mice had similar serum cholesterol concentrations (+8 and +1 %, both *p* = N.S.; data not shown) and triglyceride concentrations (data not shown) as control mice. Body weight was reduced both in female and male LI-IGF-I^−/−^ mice compared to that in control mice, and body composition measurements using DEXA revealed that both the absolute and relative amounts of total body fat were reduced in female and male LI-IGF-I^−/−^ mice compared with those in control mice (Table 1). Thus, obesity is not involved in the mechanism for increased fatty streak formation in female LI-IGF-I^−/−^ mice. There was a non-significant trend to lower lean mass in female LI-IGF-I^−/−^ mice, whereas male LI-IGF-I^−/−^ mice had significantly lower lean mass than control mice (Table 1).

**Table 1:** Body composition of female and male LI-IGF-I^−/−^ mice

	Female mice		Male mice	
	Control	LI-IGF-I^−/−^	Control	LI-IGF-I^−/−^
Body weight (g)	33.2 (1.4)	28.2 (2.0)*	41.0 (2.8)	31.3 (1.1)**
DEXA				
Body fat (g)	12.6 (1.2)	8.1 (1.5)*	15.0 (2.0)	8.8 (0.9)*
Body fat (%)	37.7 (2.5)	29.3 (3.4)*	36.3 (2.7)	29.0 (2.1)*
Lean mass (g)	18.8 (0.4)	18.0 (0.6)	23.3 (0.7)	20.7 (0.5)**

Male and female LI-IGF-I^−/−^ and control mice (*n* = 9–16/group) received an atherogenic (modified Paigen) diet from 6 months of age. At the end of the study (at 12 months of age), in vivo measurements using DEXA were performed. The DEXA scans were from the lower level of the head to the inferior level of the first tail vertebra (the maximal scan area of the DEXA). Values are given as means (SEM)

* *p* < 0.05; ** *p* < 0.01 vs. control mice using unpaired *t* tests

## Discussion

Circulating IGF-I decreases [1], and the risk of atherosclerosis increases, with advancing age. Previous studies have shown conflicting results in terms of the role of IGF-I in atherosclerosis development [16, 17, 22–24], and the specific role of liver-derived circulating IGF-I has not yet been evaluated. In the present study, we demonstrate that deficiency of liver-derived circulating IGF-I results in increased diet-induced fatty streak formation in aortic root of female but not male mice. The increased fatty streak formation in female LI-IGF-I^−/−^ mice was associated with increased macrophage accumulation (in proportion to the increased fatty streak area) as well as increased serum levels of cholesterol and IL-6. Furthermore, in female LI-IGF-I^−/−^ mice, endothelial activation was suggested by increased vascular VCAM1 mRNA levels and increased VCAM1 protein expression. Therefore, increased serum cholesterol, systemic inflammation, and endothelial dysfunction may contribute to the increased fatty streak formation in aortic root of female LI-IGF-I^−/−^ mice.

In many models of total or cell-specific knockout of IGF-I, the role of IGF-I in adult life has been difficult to evaluate due to the possible effect of the absence of IGF-I activity during pre- and postnatal development [1]. Importantly, in this study, the deficiency of endocrine, liver-derived IGF-I was induced at 3 months of age. Consequently, the mice developed normally but then underwent a maintained, selective inactivation of IGF-I in hepatocytes resulting in a 75–80 % reduction of serum IGF-I [2, 29–40]. Therefore, our data clearly show that circulating IGF-I has a vasculoprotective role in female mice, which is independent of any developmental effects. Furthermore, our results extends previous findings of reduced atherosclerosis following pharmacological administration of IGF-I using systemic infusions [22, 23], and increased atherosclerosis in genetically modified mice with a 20 % reduction of circulating IGF-I [24]. However, limitations of the present study include that apolipoprotein E-deficient mice or LDL receptor-deficient mice were not used and that, e.g., the rate of cholesterol clearance from the arterial wall as well as oxidation of cholesterol were not determined. Furthermore, other limitations are that infiltration of inflammatory cells in liver was not measured and that only one marker (VCAM1) was used to evaluate endothelial function.

We did not measure insulin sensitivity in the present study, but several previous studies have shown that deficiency of liver-derived IGF-I in mice is characterized by insulin resistance [29, 41, 42]. The LI-IGF-I^−/−^ mice have compensated insulin resistance with unchanged blood glucose level [29], reduced cardiac output [32], increased systolic blood pressure associated with increased peripheral vascular resistance [32], and signs of endothelial dysfunction associated with impaired vasorelaxation of resistance vessels [32]. In the present study, mRNA levels of eNOS in the femoral artery of female LI-IGF-I^−/−^ mice were not significantly affected. This concurs with our previous finding, which included administration of the NO-synthase inhibitor L-NAME as well as measurements of plasma nitrate, that alterations in vascular tone in LI-IGF-I^−/−^ mice are not primarily regulated by NO [32]. In a study of mice receiving pharmacological IGF-I administration by systemic infusions, the anti-atherosclerotic effect of IGF-I was NO-independent [23]. However, eNOS activity has not been evaluated in the LI-IGF-I^−/−^ mice. Therefore, we cannot exclude that a NO-mediated mechanism might be involved in the increased fatty streak formation in the LI-IGF-I^−/−^ mice.

Both IGF-I and the IGF-I receptors are expressed in the vasculature [1], indicating that IGF-I may exert its effects in an autocrine/paracrine manner. However, as observed in LI-IGF-I^−/−^ mice, IGF-I also has important endocrine actions. We previously hypothesized that the conflicting results of previous studies [16, 17, 22–24] could be due to different effects of circulating IGF-I versus locally produced IGF-I in the vasculature. Our present data suggest that liver-derived endocrine IGF-I may protect against diet-induced fatty streak formation in aortic root by improving lipid levels and endothelial function. In contrast, we previously speculated that locally produced IGF-I could potentially increase atherosclerosis by inducing VSMC migration and proliferation [1]. However, the in vitro and in vivo study by von der Thüsen suggested that VSMC-mediated effects of IGF-I could stabilize atheromatous plaques [20]. Furthermore, smooth muscle-specific IGF-1 overexpression did not alter total atherosclerotic plaque burden but resulted in a more stable atherosclerotic lesion phenotype [21]. Therefore, our present hypothesis is that liver-derived circulating IGF-I protects against atherosclerosis, whereas locally derived IGF-I in the arterial wall likely is relatively neutral in terms of atherosclerotic lesion size.

At 6 months of age, serum cholesterol was increased in female but not in male LI-IGF-I^−/−^ mice both before and 2 weeks after the start of the modified Paigen diet. It is less likely that this relatively subtle gender difference was of major importance for the sexual dimorphism in fatty streak formation. In previous studies, both male and female 4-month-old LI-IGF-I^−/−^ mice had increased serum cholesterol compared to controls [29], whereas tail-cuff systolic blood pressure was more markedly increased in female LI-IGF-I^−/−^ mice than that in male LI-IGF-I^−/−^ mice compared to that in control mice [32]. Therefore, more severely impaired systolic blood pressure in female LI-IGF-I^−/−^ mice could be one mechanism underlying the gender difference in diet-induced fatty streak formation. In the present study, IGF-I mRNA levels in the femoral artery were increased in female IGF-I^−/−^ mice, whereas there was only a non-significant tendency to an increase in male IGF-I^−/−^ mice. The importance of this finding for the observed gender differences is not clear since recent studies suggest that IGF-I in the arterial wall stabilizes atherosclerotic lesions without any effect on total atherosclerotic plaque burden [20, 21]. In summary, the mechanisms underlying the gender differences observed in the present study are not fully clear and further mechanistic studies are needed. Such studies could include estradiol or testosterone treatment of ovariectomized mice to evaluate the role of estrogen.

It has previously not been studied in detail whether the effects of IGF-I on the vasculature are sexually dimorphic in mice, but some previous studies in mice have suggested that the importance of IGF-I for life span regulation is gender specific [38, 43]. In humans, adult hypopituitary patients with severe GH deficiency and secondary low IGF-I display subclinical inflammation [44], disturbed serum lipid pattern [45], insulin resistance [46], elevated diastolic blood pressure [47], and premature atherosclerosis indicated by increased carotid intima-media thickness [48, 49]. There is some evidence that women might have more severe consequences of the hypopituitary disease than men both in terms of cardiovascular risk factors [50] and CVD morbidity and mortality [8, 51]. However, the results of epidemiological studies have not suggested any clear gender differences and the importance of possible direct effects of GH on vascular cells have not been evaluated in detail in previous studies. Therefore, our results are in line with some but not all previous findings across species that the consequences of low circulating IGF-I might differ between males and females in terms of several endpoints including atherosclerosis progression.

In the present study, female LI-IGF-I^−/−^ mice displayed increased serum cholesterol. As IGF-I exerts anti-inflammatory effects, we also measured serum cytokine levels in female mice. Serum IL-6 concentrations were increased in female LI-IGF-I^−/−^ mice both before and 2 weeks after the start of the atherogenic diet. This is in line with the previous findings in hypopituitary patients [44] and in non-diabetic subjects [52] that IGF-I is a regulator of circulating IL-6. Thus, increased systemic inflammation might contribute to increased fatty streak formation in aortic wall of female LI-IGF-I^−/−^ mice although this requires confirmation in further studies. In addition, we previously showed elevated systolic blood pressure and impaired vasorelaxation in LI-IGF-I^−/−^ mice [32]. Therefore, the increased fatty streak formation in aortic wall of female LI-IGF-I^−/−^ mice is associated with increased serum cholesterol, systemic inflammation, and signs of endothelial activation.

Body weight and percentage body fat were reduced in female as well as in male LI-IGF-I^−/−^ mice, excluding a role of obesity in the increased fatty streak formation in aortic wall of female LI-IGF-I^−/−^ mice. However, these findings are in line with previous observations that deficiency of liver-derived IGF-I results in a gradual reduction of body fat over time [1, 29, 53]. In LI-IGF-I^−/−^ mice, GH levels are compensatory increased [1, 2, 30], and it is most likely that reduced body fat in LI-IGF-I^−/−^ mice is caused by the increased GH secretion [1]. The extent to which a compensatory increase in GH levels also could affect atherosclerosis progression is not fully clear. Transgenic mice overexpressing GH have a 50- to 100-fold increase in circulating GH levels [15] and increased atherosclerosis [54], but there are no indications that a moderate increase in GH secretion would be atherogenic in mice.

In conclusion, we demonstrate that female mice with deficiency of liver-derived IGF-I display increased diet-induced fatty streak formation in the aortic root. This was associated with increased serum cholesterol and increased systemic inflammation as well as signs of endothelial activation, lipid deposition, and macrophage infiltration in the vascular wall.
